# Increased hypospadias risk by *GREM1* rs3743104[G] in the southern Han Chinese population

**DOI:** 10.18632/aging.202983

**Published:** 2021-05-07

**Authors:** Fuming Deng, Jinglu Zhao, Wei Jia, Kai Fu, Xiaoyu Zuo, Lihua Huang, Ning Wang, Huiming Xia, Yan Zhang, Wen Fu, Guochang Liu

**Affiliations:** 1 Department of Pediatric Urology, Guangzhou Women and Children’s Medical Center, Guangzhou Medical University, Guangzhou 510623, Guangdong, China; 2 Department of Pediatric Surgery, Guangzhou Institute of Pediatrics, Guangdong Provincial Key Laboratory of Research in Structural Birth Defect Disease, Guangzhou Women and Children’s Medical Center, Guangzhou Medical University, Guangzhou 510623, Guangdong, China

**Keywords:** hypospadias, GREM1, expression quantitative trait locus (eQTL), single-nucleotide polymorphism (SNP), miRNA

## Abstract

Hypospadias is a common congenital genitourinary malformation characterized by ventral opening of the urethral meatus. As a member of the bone morphogenic protein antagonist family, *GREM1* has been identified as associated with susceptibility to hypospadias in the European population. The present study was designed to elaborate on the mutual relationship between replicated single-nucleotide polymorphisms (SNPs) and hypospadias in Asia's largest case-control study in the Southern Han Chinese population involving 577 patients and 654 controls. Our results demonstrate that the *GREM1* risk allele rs3743104[G] markedly increases the risk of mild/moderate and severe hypospadias (*P<0.01*, 0.28*≤OR≤*0.66). GTEx expression quantitative trait locus data revealed that the eQTL SNP rs3743104 has more associations of eQTL SNP rs3743104 and *GREM1* targets in pituitary tissues. Additionally, Bioinformatics and Luciferase Assays show that miR-182 is identified as a suppressor for *GREM1* expression, likely through regulation of its binding affinity to rs3743104 locus. In conclusion, the *GREM1* risk allele rs3743104[G] increases hypospadias susceptibility in mild/moderate and severe cases among the southern Han population. rs3743104 regulates *GREM1* expression by altering the binding affinity of miR-182 to their locus. Collectively, this study provides new evidence that *GREM1* rs3743104 is associated with an increased risk of hypospadias. These findings provide a promising biomarker and merit further exploration.

## INTRODUCTION

Hypospadias is a common congenital genitourinary deformity characterized by a ventrally opened urethral meatus, chordee and hooded foreskin with ventral deficiency [1]. The incidence of hypospadias varies among populations, regions and ethnicities. Besides, there is an increasing trend of hypospadias in many countries. The mean prevalence was: Worldwide 20.9%, Asia 0.6–69%, Europe 18.6%, North America 34.2%, South America 5.2%, Africa 5.9%, and Australia 17.1–34.8% [2]. Hypospadias is caused by incomplete urethral fusion during gestational weeks 8 to 16 [3]. Hypospadias can be clinically divided into two subgroups according to the abnormal meatus location, namely, mild/moderate and severe hypospadias [4]. Despite rapid progress in surgical repair over recent decades, the etiology of hypospadias remains largely unknown [1, 5–7].

Genetic factors have been demonstrated to be a key element during the development of hypospadias [8, 9]. By case-control analysis, a genome-wide association study (GWAS) identified that a few genes cause genetic susceptibility, including *AR*, *SRD5A2*, *ATF3* and *BMP*. [10] Moreover, it has been reported that some SNPs correlate with hypospadias, but most relationships have been found in small studies without consistent replication [11, 12]. For instance, *GREM1* rs3743104 (NC_000015.9:g.33023985A>G) was identified by a GWAS [13] in a large European population (1,972 cases and 1,812 controls), though the results were not replicated by Chen et al. in a small Chinese population (197 cases and 933 controls) [14].

*GREM1*, which encodes a member of the bone morphogenic protein antagonist family, is located at 15q13.3, and it is reported to be closely related to a variety of malignant tumors [15]. It is worth mentioning that the epithelial-mesenchymal transition (EMT) in colon cancer cells can be suppressed by silencing of *GREM1* via short hairpin RNA (shRNA), [16] and this process is considered an essential event inducing morphogenetic changes during the development of embryos [17]. Hence, to identify the association of *GREM1* with hypospadias and the reproducibility of the link between *GREM1* rs3743104 A>G previously derived in European GWASs of hypospadias, a replication investigation was conducted on ethnically homogeneous patients (577 cases and 654 controls) from the largest Southern Han Chinese population. The relationship between *GREM1* and subtypes of hypospadias was studied. We also further demonstrated the functional relevance of *GREM1* rs3743104 on Hypospadias and the mechanism of the SNP-related MicroRNAs(miRNA) involved in regulating *GREM1* expression.

## MATERIALS AND METHODS

### Study population

A total of 557 patients undergoing repair of hypospadias diagnosed by pediatric urologists at Guangzhou Women and Children’s Medical Center from 2016 to 2018 were collected, all of whom were Southern Han Chinese. Patients with only penile curvature were excluded from this study. The cases were divided into two subtypes according to the location of the urethral meatus: mild/moderate (281 patients with coronal or glanular or shaft penis hypospadias) and severe (247 patients with penoscrotal, scrotal and perineal hypospadias) hypospadias. A total of 29 cases could not be classified because the patients underwent the first surgical repairs at other hospitals. A total of 654 healthy children who underwent a physical examination in our hospital were recruited as the healthy control group, but children who had a history of hypospadias or any experience with hypospadias repair were excluded. Each patient was informed of the study purpose, and written consent was obtained from all participants or their parents/legal guardians. In addition, ethical approval was obtained from Guangzhou Women and Children’s Medical Center in China.

### DNA extraction and genotyping

TIANamp Blood DNA kits (Catalog No. DP335-02; TIANGEN Biotech Co. Ltd., Beijing, China) were utilized to extract genomic DNA from venous blood samples of patients with hypospadias following the manufacturer’s instructions [18]. DNA concentration and purity were assessed using NanoPhotometer® N50 (Implen GmbH., Munich, Germany). An ABI-7900 real-time quantitative PCR instrument (Applied Biosystems, Foster City, CA, USA) was used to amplify genomic DNA, which was subsequently subjected to TaqMan genotyping for the *GREM1* rs3743104 [19–21]. PCR reactions were run in duplicates in a total volume of 5 μl containing 1 μl (40 ng) genomic DNA, 0.04 μl TaqMan® SNP Genotyping Assays (Catalog No: 4351379, C_180222_20, Thermo Fisher, Grand Island, NY, USA), 2.5 μl TIANexact genotyping qPCR PreMix(Probe) (Catalog No. FP211-02; TIANGEN Biotech Co. Ltd., Beijing, China) and 1.46 μl DNase/RNase-free H_2_O with 98% concordance rate for genotype calls. Moreover, 10% of DNA samples were selected at random for another genotyping analysis. All of the replicated samples were entirely consistent to ensure that the data were accurate [22].

### Bioinformatics analysis

To further investigate the potential impact of *GREM1* rs3743104, we used TargetScan (http://www.targetscan.org) and MirSNP databases (http://bioinfo.bjmu.edu.cn/mirsnp/search/) to identify that rs3743104 at the 3′-UTR of *GREM1* might contain a microRNA (miRNA) binding site. TargetScan is a web server that predicts biological targets of microRNAs (miRNAs) by searching for the presence of sites that match the seed region of each miRNA. MirSNP (http://bioinfo.bjmu.edu.cn/mirsnp/search/) allows for searching miRNA-related SNP sites.

The majority of GWAS risk loci are present in noncoding genomic regions with many gene regulatory signals, suggesting that most disease-causing SNPs exert their phenotypic effects by altering gene expression [23, 24]. *GREM1* rs3743104 is in the 3’-UTR of *GREM1* (noncoding genomic regions). Human cell population-based or tissue-based expression quantitative trait locus (eQTL) mapping studies, such as Genotype-Tissue Expression Project (GTEx), have provided an unprecedented opportunity to explore context-dependent regulatory patterns surrounding these loci [25]. To examine the eQTL SNP *GREM1* rs3743104 in different tissues, we downloaded significant SNP-gene associations from GTEx V4 from the GTEx portal (http://www.gtexportal.org/), which contains eQTL mapping results for expression of different genotypes in different tissues.

### Luciferase assays

The wild-type GREM1 dual reporting vector was obtained by the GREM1 PCR product and the psiCHECK™-2 (Catalog No: C8011, Promega, Madison, WI, USA) vector though digested with XhoI/NotI (XhoI: Catalog No: D1094A, TaKaRa Biotech Co. Ltd., Beijing, China; NotI: Catalog No: D1166A, TaKaRa Biotech Co. Ltd., Beijing, China). The GREM1 mutant sites vector was successfully constructed by PCR and DpnI enzyme (Catalog No: R6231, Promega, Madison, WI, USA). Sequences of the wild-type GREM1 dual reporting vector and the GREM1 mutant sites vector were shown in Supplementary Table 1. Each of the DNA products was separately subcloned into a luciferase reporter plasmid at the downstream of luciferase coding region (psiCHECK2; Promega, Madison, WI, USA) and the sequence validity was confirmed by Sanger sequencing. Next, 2×10^4^ HEK293T cells were seeded into each well of 24-well plate and simultaneously transfected with respective construct, either miR-182, miR-212, miR-221, miR-3128 mimics and correspond MicroRNA inhibitor(Sequences of miR-182, miR-212, miR-221, miR-3128 mimics were shown in Supplementary Table 2) using lipofectamine 2000 (Catalog No: 116680119, Invitrogen, Carlsbad, CA, USA). After being cultured for 48 h, luciferase activity of the cells was measured with a Dual-Luciferase Reporter Assay Kit (Catalog No: E1910, Promega, Madison, WI, USA) according to the manufacturer’s protocol.

### Statistical analysis

GraphPad Prism version 8 (GraphPad Software, Inc., La Jolla, CA, USA) and SAS 9.4 software (SAS Institute Inc., Cary, NC, USA) was used to perform statistical analyses in this study. A chi-squared goodness-of-fit test was conducted to examine the consistency between the genotype frequencies of the controls and Hardy–Weinberg equilibrium (HWE). The SNP was analyzed for association with the disease by comparing the risk of allele frequency (allelic test) in patients and controls as well as other tests using PLINK 1.9 (test of dominant and recessive models, genotype test) [26]. Association stratified by subphenotype was analyzed by comparing cases with a certain subphenotype with controls. A P-value of 0.05 was considered statistically significant.

## RESULTS

### Association between GREM1 polymorphism rs3743104 and hypospadias susceptibility

In the present study, 525 of 557 cases and 594 of 654 controls were successfully genotyped. Table 1 shows the crude and adjusted odds/P values for the GREM1 polymorphism rs3743104 and hypospadias susceptibility. The frequency distribution of Hardy-Weinberg equilibrium (P_HWE=0.32). Comparison between Hypospadias patients and controls revealed that AA and AG genotypes were significantly higher in controls. In contrast, the GG genotype was more frequent in patients (65.71% in case versus 32.57% in controls, P=3.25×10^-12^/Adjusted P=1.99×10^-12^). Consistently, the G allele was more prevalent among patients (77.28%) compared to control group (55.12%). The GREM1 risk allele rs3743104[G] was associated with an increased risk of hypospadias. Our results also illustrated that GREM1 rs3743104[G] conferred increased risk to hypospadias under homozygote comparison based on different genotypic models, including dominant and recessive models (GG+AG versus AA: Adjusted OR=3.07, Adjusted P=1.32×10^-06^;GG versus AG+AA: Adjusted OR=4.43, Adjusted P=1.62×10^-28^).

**Table 1:** Association between *GREM1* polymorphism rs3743104 and hypospadias susceptibility.

**Genotype**	**Case** **(n=557)**	**Control** **(N=654)**	**Crude OR (95% CI)**	***P***	**Adjusted OR (95% CI)^1^**	***P*^1^**
AA	30 (53.86)	86 (13.15)	Ref	1.00	Ref	1.00
AG	129 (23.16)	295 (45.11)	1.25(0.79-1.99)	0.34	1.35(0.92-2.22)	0.24
GG	366 (65.71)	213 (32.57)	4.93(3.15-7.72)	**3.25×10-12**	5.30(3.33-8.43)	**1.99×10-12**
Genotypic model			NA	**1.91×10-27**	NA	**1.63×10-27**
Dominant model (GG+AG versus AA)	495/30	508/86	2.79(1.81-4.31)	**3.44×10-06**	3.07(1.95-4.83)	**1.32×10-06**
Recessive model (GG versus AG+AA)	366/159	213/381	4.12(3.21-5.29)	**1.73×10-28**	4.43(3.41-5.77)	**1.62×10-28**

Values are shown as number (%); AA, homozygous protective; AG, heterozygous; GG, homozygous risk for rs3743104; OR (95% CI), odds ratio and confidence interval; ^1^Adjusted for age with omission of the corresponding stratification factor; P value surpassing statistical significance (0.05) were boldfaced.

### Subtype analysis of the relationship between GREM1 polymorphism rs3743104 and hypospadias risk

Hypospadias subtypes are classified according to the meatus location, including mild/moderate and severe hypospadias. The *GREM1* risk allele rs3743104[G] notably increased the risk of mild/moderate and severe hypospadias (*P<0.01,* 0.28*≤OR≤*0.66, Table 2).

**Table 2:** Stratification analysis for the association between *GREM1* risk allele rs3743104[G] and hypospadias susceptibility (by subgroup).

rs3743104	**Case**		**Control**	**Case-only**			**Severe-only**			**Mild/moderate -only**	
	**Severe**	**Mild/moderate**		***P***	***OR*(CI95)**		***P***	***OR*(CI95)**		***P***	***OR*(CI95)**
	0.1533	0.2065	0.3931	**<0.01**	0.66(0.47-0.92)		**<0.01**	0.28(0.21~0.36		**<0.01**	0.40(0.32~0.51)

Case-only: All the patients with hypospadias. Severe-only: patients with penoscrotal, scrotal and perineal hypospadias. Mild/moderate-only: patients with coronal or glanular or shaft penis hypospadias. P value surpassing statistical significance (0.05) were boldfaced.

### Regulatory element analysis of miRNAs targeting rs3743104

As rs3743104 is located at the 3′-UTR of *GREM1*, it is suspected that rs3743104 may affect the binding of miRNAs. Using the TargetScan and MirSNP databases, we identified that hsa-miR-182-5p, hsa-miR-212-5p, hsa-miR-221-5p and hsa-miR-3128 might bind to a site at the *GREM1* 3'-UTR (Table 3). In fact, based on TargetScan and MirSNP predictions, different alleles of rs3743104 would affect the binding of microRNAs to *GREM1*.

**Table 3:** rs3743104 regulates *GREM1* though miRNAs.

**SNP**	**Gene**	**miRNA**	**Effect**	**Allele**
rs3743104	*GREM1*	hsa-miR-182-5p	create	G/A
		hsa-miR-212-5p	create	G/A
		hsa-miR-221-5p	break	G/A
		hsa-miR-3128	break	G/A

The microRNA (miRNA) binding site rs3743104 in the 3′ UTR of *GREM1* were predicted by TargetScan (http://www.targetscan.org) and MirSNP databases (http://bioinfo.bjmu.edu.cn/mirsnp/search/). Create means that the occurrence of the SNP leads to a miRNA binding site in the mRNA. Break means that the occurrence of the SNP causes the loss of miRNA binding site.

### GTEx eQTL analysis of rs3743104 and GREM1 expression

**Figure 1:**
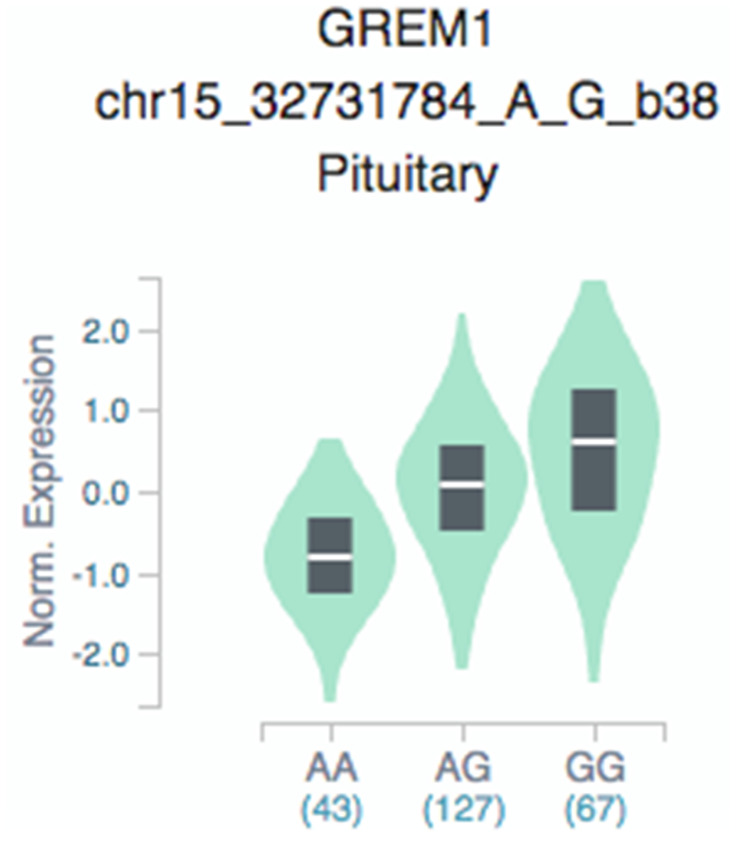
**GTEx eQTL analysis of rs3743104 and *GREM1* expression in pituitary tissues.** GTEx: Genotype-Tissue Expression, *GREM1* rs3743104 (chr15:33023985;GRCh37/hg19, A/G); AA, homozygous protective; AG, heterozygous; GG, homozygous risk for rs3743104.

Loci in which expression level is influenced by specific genetic variation are termed expression quantitative trait loci (eQTL), which refers to genetic variants that affect expression in an allele-specific manner, with implications on an underlying mechanism [27, 28]. We sought to illustrate SNP associations with expression of *GREM1* in different tissues, and GTEx eQTL data revealed more associations of different genotypes of eQTL SNP rs3743104 and *GREM1* targets in pituitary tissues (Figure 1). Compared with the *GREM1* rs3743104 GG genotype, the AG/AA genotype resulted in reduced expression of *GREM1*.

### Hsa-miR-182-5p targeted rs3743104

**Figure 2:**
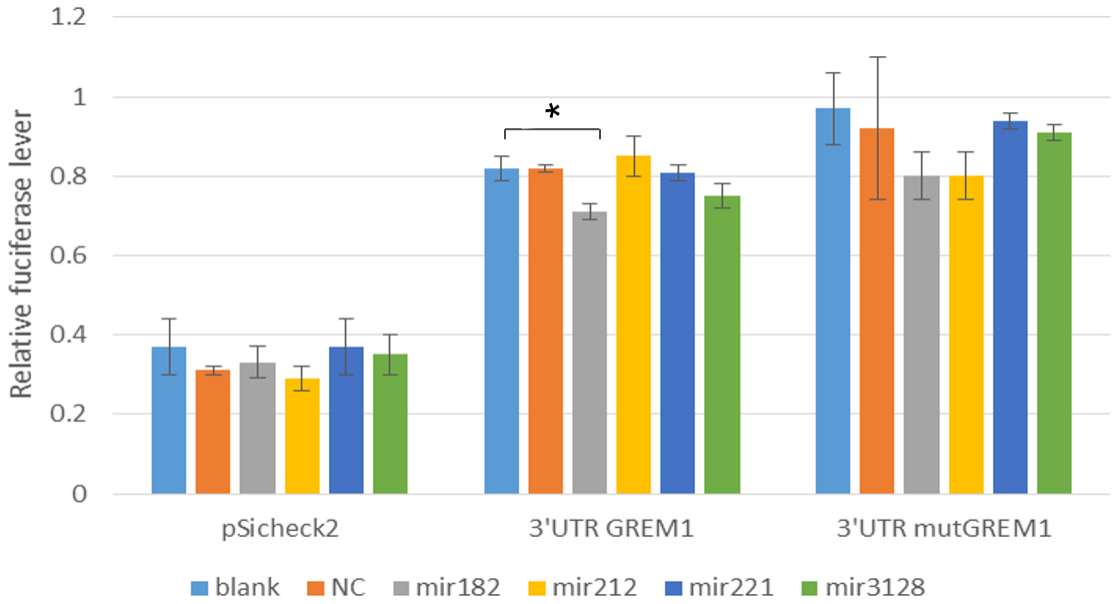
**Luciferase activities in HEK293T cells transfected with respective construct, either miR-182, miR-212, miR-221, miR-3128 mimics and correspond MicroRNA inhibitor.** NC: Negative control. Comparing each group with the blank group, *:P=0.0478, *P<0.05* significant difference.

To further investigate how rs3743104 regulates upstream GREM1, we identified that hsa-miR-182-5p, hsa-miR-212-5p, hsa-miR-221-5p and hsa-miR-3128 might bind to a site at the GREM1 3'-UTR (Table 3). We identified that hsa-miR-182-5p might target a miRNA binding site at the 3′-UTR of RPA1, using TargetScan. Luciferase reporter assays showed that the luciferase activity of GREM1 3′-UTR was significantly reduced in the 293T cells transfected with miR-182 mimics compared to the control group in a dose-dependent manner (Figure 2). The data for Figure 2 were shown in Supplementary Table 3.

## DISCUSSION

Hypospadias is a common congenital urethral anomaly. In previous studies, [13] it was confirmed that SNP rs3743104 is associated with hypospadias susceptibility. Kojima et al. [29] replicated 17 SNPs of *HAAO* identified by European GWASs [13] and showed that only one SNP was strongly associated with hypospadias susceptibility in the Japanese population, indicating different allele frequencies of causative variants for hypospadias among different ethnic groups. However, according to a replication study, rs3743104 had no relationship with hypospadias susceptibility in a small Chinese population. Comparing to the previous GWAS study, the recruited population and region were different. Although the populations of the previous Chinese GWAS study and our study belonged to the Han population, the distribution of the population was different from that our population mainly located in the Southern area of China. Except for genetic factors, the environmental factor is also contributing to hypospadias. That may be the reason why the result was different from the research of Chen et al. [14] Thus, another replication study was conducted on a larger Southern Han Chinese population to assess the association of SNP rs3743104 with hypospadias susceptibility.

This is the largest Chinese population-based study to examine the correlation of *GREM1* genetic polymorphisms with hypospadias risk, enrolling 557 cases and 645 unrelated controls. In this study, the associated of *GREM1* rs3743104 with hypospadias was replicated (Table 1). Further analysis of subclinical manifestations clarified SNP rs3743104 and revealed associations with mild/moderate and severe hypospadias (Table 2). Given that rs3743104 is located at the 3′-UTR of *GREM1*, we determined a series of miRNAs predicted to bind to this region, suggesting that rs3743104 has a regulatory effect on expression of *GREM1* (Table 3). eQTL analysis suggests that *GREM1* rs3743104 may reduce androgen production by the hypothalamic-pituitary-gonadal axis (HPG axis, Figure 1). The HPG axis in males has a central role in developing the genitalia, including spermatogenesis and testosterone production [30]. The testosterone of hypospadias patients was significantly lower than that of the normal control population after human chorionic gonadotrophin stimulation [31]. Given that rs3743104 is located at the 3′-UTR of *GREM1*, we provided a series of experimental evidence showing that rs3743104 has a regulatory effect on the expression of upstream *GREM1*, which thereby might contribute to the progression of Hypospadias. we identified that miR-182 could target the seeding region spanning rs3743104 at the 3′-UTR of *GREM1*, which suppresses the transcription of *GREM1*. miR-182 has been reported as a biomarker for kidney injury and bladder cancer, and a regulatory miRNA binding to 3′-UTR of gene targets to the progression of diseases [32, 33]. It's worthy of further work that how miR-182 is regulated. Our study has some strengths. In addition to the largest sample size in Asia, our discovery withstood a strict threshold for statistical significance corrected by multiple tests. The clinical associations remained significant after adjusting for known prognostic factors.

*GREM1* is located at 15q13.3 and encodes a member of the bone morphogenic protein antagonist family. G*REM1* has been reported to have a close relationship with a variety of malignant tumors in recent years. For example, Gu et al. [15] reported that overexpression of *GREM1* in osteosarcoma cells *in vitro* and *in vivo* suppresses migration, invasion, angiogenesis and proliferation. It has also been shown that SNPs near *GREM1* and *SCG5* are significantly related to increased risk of colorectal cancer (CRC) [34]. Jang et al. [35] mentioned that *GERM1*-expressing fibroblasts were closely associated with lower invasiveness and better prognosis in CRC, suggesting that stromal GREM1 may represent a novel target for the treatment of CRCs and a potential biomarker. Neckmann et al. [36] showed that GREM1 is related to metastasis and poor prognosis in patients with ER-negative breast cancer and that it is a potential target for treatment. These studies reveal that GREM1 plays a vital role in tumorigenesis and can serve as a biomarker for prognosis prediction and therapeutic targets. It is worth mentioning that EMT in colon cancer cells can be suppressed by silencing of *GREM1* with shRNA, [16] and EMT is considered a critical event inducing morphogenetic changes during the process of embryonic development [17]. Church et al. [37] found that among *GREM1*-knockout mice with a C57BL/6 background, most died in a short period of time after birth due to lung defects and kidney dysplasia. Wang [38] et al. revealed a significant correlation between *GREM1* polymorphism rs1258763 and the risk of nonsyndromic orofacial clefts, and Viena CS [39] found that interaction between *NTN1* and *GREM1* may be associated with the pathogenesis of nonsyndromic cleft lip with or without cleft palate. It was also reported that *GREM1* inhibits BMP2-mediated differentiation of MSCs to osteoblasts [40]. In human esophageal squamous cell carcinoma, EMT can be promoted by *GREM1* derived from mesenchymal stromal cells [41]. In mice, hypospadias can be induced by dibutyl phthalate (DBP), which is capable of inhibiting EMT in urethral epithelial cells and blocking fusion of the urethral meatus via oxidative stress [42]. Thus, it was hypothesized that *GREM1* might have an impact on urethral development through EMT. Nevertheless, the biological mechanism underlying the correlation between *GREM1* polymorphism and hypospadias sensitivity remains unclear.

This study demonstrated the association of rs3743104 with hypospadias in mild/moderate and severe cases, whereas previous research failed to allocate patients into subgroups. In addition, this study has some limitations. First, the effect of gene-environment interactions was not analyzed. Second, more precise ORs should be adjusted based on fetal exposure. Third, in-depth analyses are warranted to explore the biological mechanisms of *GREM1* rs3743104 and hypospadias sensitivity.

## CONCLUSION

In summary, *GREM1* risk allele rs3743104[G] increase susceptibility to hypospadias in mild/moderate and severe cases from the Southern Han Chinese population and regulates *GREM1* expression by altering the binding affinity of miR-182 to their locus. Further studies are required to understand the biological mechanism by which variations in *GREM1* contribute to the pathogenesis of hypospadias.

## Supplementary Material

Supplementary Tables
