# Supplementary material for: Increased hypospadias risk by GREM1 rs3743104[G] in the southern Han Chinese population
Source: Aging (Albany NY). 2021 May 7;13(10):13898–908. doi: 10.18632/aging.202983 (PMC8202882; doi:10.18632/aging.202983)
Supplement: Supplementary Tables [file aging-13-202983-s001.pdf]

SUPPLEMENTARY TABLES

Supplementary Table 1. Sequences of the wild-type GREM1 dual reporting vector and the *GREM1* mutant sites vector.

Name	Sequences
**The wild-type *GREM1* dual reporting vector**	gccaaatccaggtgcacccagcatgtcctaggaatgcagccccaggaagtcccagacctaaaacaaccagattcttacttggcttaaacctagaggccagaagaaccccccagctgcctcctgg<br>caggagcctgcttgtgcgtagttcgtgtgcatgagtgtggatgggtgcctgtgggtgtttttagacaccagagaaaacacagtctctgctagagagcactccctattttgtaaacatatctgctttaat<br>ggggatgtaccagaaacccacctcaccccggctcacatctaaaggggcggggccgtggtctggttctgactttgtgtttttgtgccctcctggggaccagaatctcctttcggaatgaatgttcatg<br>gaagaggctcctctgagggcaagagacctgttttagtgctgcattcgacatggaaaagtccttttaacctgtgcttgcatcctcctttcctcctcctcctcacaatccatctcttcttaagttgatagtgac<br>tatgtcagtctaatctcttgtttgcca**a**ggttcctaaattaattcacttaaccatgatgcaaatgtttttcattttgtgaagaccctccagactctgggagaggctggtgtgggcaaggacaagcaggat<br>agtggagtgagaaagggagggtggagggtgaggccaaatcaggtccagcaaaagtcagtagggacattgcagaagcttgaaaggccaataccagaacacaggctgatgcttctgagaaagt<br>cttttcctagtatttaacagaacccaagtgaacagaggagaaatgagattgccagaaagtgattaactttggccgttgcaatctgctcaaacctaacaccaaactgaaaacataaatactgaccactc<br>ctatgttcggacccaagcaagttagctaaaccaaaccaactcctctgctttgtccctcaggtggaaaagagaggtagtttagaactctctgcataggggtgggaattaat
**The *GREM1* mutant sites vector**	Gccaaatccaggtgcacccagcatgtcctaggaatgcagccccaggaagtcccagacctaaaacaaccagattcttacttggcttaaacctagaggccagaagaaccccccagctgcctcctgg<br>caggagcctgcttgtgcgtagttcgtgtgcatgagtgtggatgggtgcctgtgggtgtttttagacaccagagaaaacacagtctctgctagagagcactccctattttgtaaacatatctgctttaat<br>ggggatgtaccagaaacccacctcaccccggctcacatctaaaggggcggggccgtggtctggttctgactttgtgtttttgtgccctcctggggaccagaatctcctttcggaatgaatgttcatg<br>gaagaggctcctctgagggcaagagacctgttttagtgctgcattcgacatggaaaagtccttttaacctgtgcttgcatcctcctttcctcctcctcctcacaatccatctcttcttaagttgatagtgac<br>tatgtcagtctaatctcttgtttgcca**a**ggttcctaaattaattcacttaaccatgatgcaaatgtttttcattttgtga<br>WT: a<br>MT: g<br>agaccctccagactctgggagaggctggtgtgggcaaggacaagcaggatagtggagtgagaaagggagggtggagggtgaggccaaatcaggtccagcaaaagtcagtagggacattg<br>cagaagcttgaaaggccaataccagaacacaggctgatgcttctgagaaagtcttttcctagtatttaacagaacccaagtgaacagaggagaaatgagattgccagaaagtgattaactttggcc<br>gttgcaatctgctcaaacctaacaccaaactgaaaacataaatactgaccactcctatgttcggacccaagcaagttagctaaaccaaaccaactcctctgctttgtccctcaggtggaaaagagag<br>gtagtttagaactctctgcataggggtgggaattaat
***GREM1*-WT-XhoIF**	5'ccgctcgagGCCAAATCCAGGTGCACCCAGCATGTCCTA 3'
***GREM1*-WT-NotIR**	5'ataagaatgcggccgcATTAATTCCCACCCCTATGCAGAGAGT 3'
***GREM1*-mutF**	5'CTAATCTCTTGTTTGCCAGGGTTCCTAAATTAATTCACTTAACCATGATGCAAATG 3'
***GREM1*-mutR:**	5'TTAATTTAGGAACCCTGGCAAACAAGAGATTAGACTGACATAGTCACTATCAACTT 3'

The yellow area in sequence of the wild-type *GREM1* dual reporting vector indicates the rs3743104 site; The sequences within the range of upstream 1000bp to downstream 1000bp of *GREM1* rs3743104 are display in the table; The yellow area in sequence of the *GREM1* mutant sites vector indicates the area to be mutated.

Supplementary Table 2. Sequences of miR-182, miR-212, miR-221, miR-3128 mimics.

Name	Accession	Sequence
hsa-miR-182-5p mimics	MIMAT0000259	UUUGGCAAUGGUAGAACUCACACU
hsa-miR-212-5p mimics	MIMAT0022695	ACCUUGGCUCUAGACUGCUUACU
hsa-miR-221-5p mimics	MIMAT0004568	ACCUGGCAUACAAUGUAGAUUU
hsa-miR-3128 mimics	MIMAT0014991	UCUGGCAAGUAAAAAACUCUCAU

Supplementary Table 3. Luciferase activities in HEK293T cells transfected with respective construct, either miR-182, miR-212, miR-221, miR-3128 mimics and correspond MicroRNA inhibitor.

mean	blank	NC	mir182	mir212	mir221	mir3128
pSicheck2	0.37±0.07	0.31±0.01	0.33±0.04	0.29±0.03	0.37±0.07	0.35±0.05
3'UTR GREM1	0.82±0.03	0.82±0.01	0.71±0.02	0.85±0.05	0.81±0.02	0.75±0.03
3'UTR mutGREM1	0.97±0.09	0.92±0.18	0.80±0.06	0.80±0.06	0.94±0.02	0.91±0.02

Data are shown as mean ± SD; NC: Negative control; Comparing each group with the blank group.
